# Middle East Pain Registry (MEPAIN): Feasibility Study of Chronic Pain Registry and Pilot Phase Results: Middle East Pain Registry (MEPAIN)

**DOI:** 10.31661/gmj.v14i.3807

**Published:** 2025-04-29

**Authors:** Habib Zakeri, Mohammad Radmehr, Aliasghar Karimi, Leala Montazeri, Pegah Pedramfard, Parisa Mahdiyar, Farnaz Hemati, Atiyeh Ebrahimi, Sogand Sadeghi, Saba Moalemi

**Affiliations:** ^1^ Research Center for Neuromodulation and Pain, Shiraz University of Medical Sciences, Shiraz, Iran; ^2^ Mid Cheshire NHS Foundation Trust. Leighton Hospital, Middlewich Road, Crewe, Cheshire CW1 4QJ, UK; ^3^ Mazandaran University of Medical Sciences, Sari, Iran; ^4^ Student Research Committee, Shiraz University of Medical Sciences, shiraz, Iran

**Keywords:** Pain, Registry, Low Back Pain, Chronic Pain, Middle East

## Abstract

**Background:**

Chronic pain is a significant public health concern due to its long-term disabling effects. To support systematic data collection and improve patient management, the Middle East Pain Registry (MEPAIN) was developed. This study outlines the registry’s design, evaluates its feasibility, and presents initial findings from its pilot phase.

**Materials and Methods:**

MEPAIN was launched on July 21, 2024, with data collected via the Zigorat® software platform through January 22, 2025 for this pilot study. Each patient record included demographic details, pain characteristics (pattern, location, intensity), physical exam findings, imaging results, diagnoses, interventions, and follow-up data.

**Results:**

A total of 3,903 patients were registered during the six-month pilot. The cohort was 59.5% female, with a mean age of 53.5 ± 14.8 years; 50.2% were Iranian and 49.5% Omani. Lumbar radiculopathy was the most frequent diagnosis. Osteoarthritis and carpal tunnel syndrome predominated among females, while lumbar radiculopathy and discogenic pain were more common in younger patients. Iranians reported higher pain intensity during exacerbations, while Omanis showed greater prevalence of discogenic pain, spinal stenosis, carpal tunnel syndrome, and failed back surgery syndrome. Paresthesia was the most frequently reported symptom, and transforaminal epidural steroid injection was the most common procedure performed.

**Conclusion:**

The MEPAIN registry successfully captures comprehensive clinical and procedural data on patients with chronic pain in the Middle East. It offers a robust platform for clinical evaluation and research, supporting future efforts to tailor pain management strategies in regional populations.

## Introduction

Chronic pain is a major global health issue, contributing significantly to disability and impairing daily activities and work productivity. [1] The global burden of chronic pain is not only substantial but also escalating. For instance, in China, the direct medical costs for chronic pain management doubled within just four years,[2] highlighting the growing economic impact of this condition. Additionally, chronic pain results in the loss of more than 50 million workdays annually, further exacerbating its societal burden. In the United States alone, the annual economic cost of pain exceeds US $600 billion, surpassing the costs associated with many fatal diseases. [3] Globally, low back pain stands out as the leading cause of years lived with disability, with the Middle East and North Africa being the region’s most heavily burdened by this condition.[4]

While chronic pain is often linked to injuries or biological diseases, it is important to recognize that psychological co-morbidities can also play a significant role. These co-morbidities may either contribute to the development of chronic pain or influence its severity.[5][6] However, chronic pain is not merely a secondary symptom of other conditions; it is a distinct health issue with its own definition and diagnostic criteria.[7][8] As a common and multi-factorial condition, chronic pain profoundly impacts an individual’s social role, quality of life, and financial stability, while also imposing a significant economic burden on society as a whole. [9] Given its widespread impact, there is an urgent need for further research to improve chronic pain management strategies.

To advance research in this field, access to comprehensive patient data is essential. Researchers require databases of patients with chronic pain to conduct thorough investigations efficiently. [10][11][12] This is where patient registries or electronic medical records become invaluable tools. A patient registry is an organized system that uses observational study methods to collect uniform data, enabling the assessment of specific outcomes in populations with particular diseases or conditions. [12] Although patient registries have certain limitations, they are highly effective in evaluating the efficacy and safety of therapeutic methods across diverse patient populations and clinical settings.

Due to pilot phase, in line with this need, the Nab Pain Clinic (NPC) which is affiliated with the Research Center for Neuromodulation and Pain at Shiraz University of Medical Sciences in Shiraz, Iran. This center provides a range of pain management treatments for patients with various chronic pain disorders, particularly serving the Middle East region.

We initiated the development of a registry to document medical records related to chronic pain. This manuscript outlines the development, feasibility evaluation, and pilot phase results of the Middle East Pain Registry (MEPAIN). Additionally, it describes the structure and content of the registry, as well as the characteristics of the patients.

## Materials and Methods

**Figure-1:**
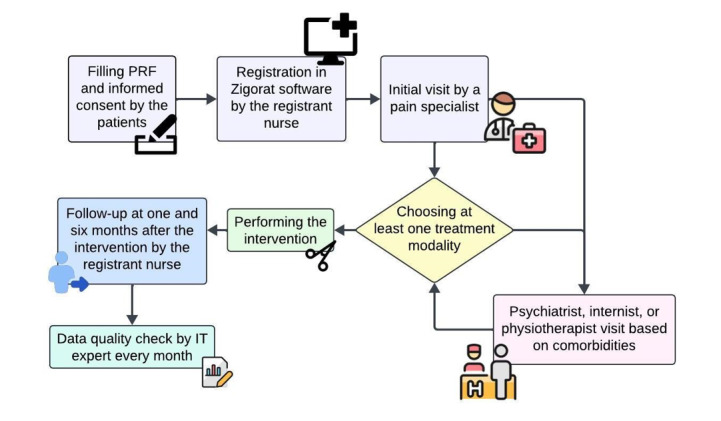
Figure 1. The flowchart outlines a patient management process where patients provide consent and are registered by a nurse, followed by an initial consultation with a pain specialist who selects a treatment modality. The patient may see additional specialists based on comorbidities, with follow-up conducted at 1 and 6 months, and regular data quality checks performed monthly.

**Table:** Table1. Demographic characteristics and comorbidities of the patients.

**Variable**		**Frequency**
**Total**		3903
	Below 40	856 (21.9%)
**Age group**	40-60	1694 (43.4%)
	Above 60	1353 (34.7%)
**Gender**	Male	1581 (40.5%)
	Female	2322 (59.5%)
	Normal (18-24.9)	1126 (28.8%)
**BMI**	Overweight (25-29.9)	1501 (38.5%)
	Obese (30 and above)	865 (22.2%)
	Not reported	411 (10.5%)
	Iran	1959 (50.2%)
	Oman	1930 (49.45%)
**Nationality**	Iraq	9 (0.23%)
	Bahrain	4 (0.1%)
	Qatar	1 (0.02%)
	Hypertension	1112 (28.5%)
	Diabetes mellites	758 (19.4%)
	Hyperlipidemia	734 (18.8%)
	Ischemic heart disease	376 (9.6%)
	Hypothyroidism	267 (6.8%)
	Renal diseases	172 (4.4%)
**Past Medical History**	Pulmonary disease	107 (2.7%)
	Neuromuscular diseases	85 (2.2%)
	Rheumatoid arthritis	82 (2.1%)
	Stroke	48 (1.2%)
	Hyperthyroidism	34 (0.9%)
	Cancer	33 (0.8%)
	Osteoarthritis	9 (0.2%)

**Table:** Table2. Comparison of pain features and diagnoses between demographic variables

**Variables**	**Total**		**Gender**			**Age**				Nationality ***	
		**male**	**female**	**P-value**	**Below 40**	**40-60**	**Above 60**	**P-value**	**Iran**	**Oman**	**P-value**
Pain duration (months)*	36.5± 1.18 [Min= 3, Maxi=168]	36.33 (34.46- 38.21)	36.63 (35.10- 38.16)	0.810	35.15 (32.68- 37.61)	36.56 (34.77- 38.34)	37.33 (35.26- 39.40)	0.643	35.48 (33.83 - 37.13)	37.57 (35.86 - 39.28)	0.086
Current pain score*	5.8 ±2.6	5.80 ±2.67	5.85 ±2.7	0.653	5.95±2.69	5.77±2.69	5.8±2.68	0.412	5.87±2.75	5.78±2.62	0.323
Pain score (best condition)*	5±2.5	4.92 ±2.58	4.95 ±2.58	0.858	5.08±2.62	4.9±2.57	4.86±2.56	0.329	4.9±2.65	4.97±2.51	0.427
Pain score (worst condition)*	8.1±1.7	8.08 ±1.75	8.14±1.69	0.298	8.16±1.65	8.05±1.75	8.12±1.76	0.165	8.26±1.7	7.94±1.75	**<0.01**
Pain diagnosis**											
**Lumbar radiculopathy**	1215 (31.13)	502(31.75)	713(30.71)	0.489	259(30.26)	528(31.17)	428(31.63)	0.792	611(31.19)	599(31.04)	0.918
**Osteoarthritis**	933(23.9)	331(20.94)	602(25.93)	**<0.01**	0(0)	506(29.87)	427(31.56)	**<0.01**	465(23.74)	465(24.09)	0.794
**Discogenic pain**	681(17.45)	287(18.15)	394(16.97)	0.338	161(18.81)	292(17.24)	228(16.85)	0.476	298(15.21)	380(19.69)	**<0.01**
**Spinal stenosis**	358(9.17)	133(8.41)	225(9.69)	0.175	22(2.57)	134(7.91)	202(14.93)	**<0.01**	148(7.55)	208(10.78)	**<0.01**
**Carpal tunnel syndrome**	110(2.82)	16(1.01)	94(4.05)	**<0.01**	13(1.52)	60(3.54)	37(2.73)	**0.014**	31(1.58)	78(4.04)	**<0.01**
**Failed back surgery syndrome**	59(1.51)	18(1.14)	41(1.77)	0.115	3(0.35)	23(1.36)	33(2.44)	**<0.01**	43(2.19)	16(0.83)	**<0.01**

* Variables presented as mean±SD with 95% CI. ** Variable frequencies presented as numbers (%). *** Other nationalities were excluded from comparative analysis due to n<10. **** Independent t-test or ANOVA with Tukey post hoc analysis was used.

MEPAIN was developed with the aim of systematical recording of patient data to better understand the prevalence and characteristics of chronic pain in the region of the West of Asia and also to aid us in evaluating and comparing the efficacy of various available treatment options. In this study, we have designed a pilot phase of MEPAIN and reported the prevalence, pattern, clinical findings, and treatment methods of chronic pain among the patients referred to our clinic.

We defined our objectives as demographic characteristics, pain features, physical exam, and imaging findings, and implemented interventions.

### Study Population

This study included all patients referred to NPC with chronic pain, defined by the International Classification of Diseases, 11th edition (ICD-11), as pain persisting for more than three months, regardless of the underlying cause.[13] The patients’ agreement to participate in this study was necessary and they were free to withdraw from the study at any time. All the patients who experienced pain for less than three months were excluded from the study.

### Description of the Region

The Middle East consists of 18 countries, which include Bahrain, Cyprus, Egypt, Iran, Iraq, Israel, Jordan, Kuwait, Lebanon, Oman, Palestine, Qatar, Saudi Arabia, Syria, Turkey, the United Arab Emirates, and Yemen. ("How Many Countries Are There In The Middle East?" 2020) There is no documented evidence about the prevalence and pattern of chronic pain in this region.

### Research Team

The team for this project included two pain specialists, an internist, a psychiatrist, a physiotherapist, and two nurses. The nurses were assigned in charge of data collection and patient registration. Through a session, our pain specialist, who was the principal investigator [14] and responsible for approving the final diagnoses, explained the aim of the project and provided a thorough explanation of the items of the case report forms (CRF) to the registrar nurses. They were trained to guide the patients and their caregivers through each question of the questionnaire and also to enter patient data into the system under the supervision of the PI.

Also, an internist, a psychiatrist, and a physiotherapist helped the PI manage the patients. Nurses registered the patients’ data in Zigorat® software. The PI was available for consultation in case of challenging situations with the PRF or missing data. All data were simultaneously recorded into an NPC’s server.

### Data Collection

The data collection flow chart is depicted in figure-1. In phase one, the data were collected in a paper CRF, which was exclusively designed for this study. It consists of two main sections. The first section collected demographic data, medical history, medication use, and pain characteristics, completed by patients or caregivers to minimize bias from nursing staff.

We inquired about the demographic characteristics that have shown a role in chronic pain parameters in previous studies, including age, gender, nationality, and BMI.[15][16][17] The second section, filled by our pain specialist, is composed of a physical examination diagnostic imaging finding, and future treatment plan. Our pain specialist did all the physical examinations to avoid the difference in skills as a confounding factor.

Pain intensity, as the indicator of pain’s sensory component [18], was reported through the visual analog scale (VAS), which is a patient-reported pain rating scale recorded by choosing one point along a 10-cm line that represents the spectrum of pain severity. So, it is numerically reported as a number between one and ten [19]. The patients were asked to report their pain intensity score at their best and worst condition, as well as their current pain score.

The location and bodily distribution of the pain, as important pain domains, were assessed through the pain drawing scale. The patients were given directions to mark the regions where they experienced pain. [18]

Both pain drawing and visual analog scales are valid and reliable tools for assessing pain location and intensity, respectively [20][21].

### Initial Visit and Follow-up

After registration, the patients were visited by the pain specialist, and then by an internist, a psychiatrist, or a physiotherapist based on their underlying disease. Different treatment options were discussed with the patients and the most suitable one was chosen according to the relevant guidelines and patient preferences. One and six months after the intervention, the registrar nurses follow up with the patients or their caretakers in person or by telephone to assess their condition using the VAS and inquire about any adverse events, such as hematoma.

### Patient Report Form Validity Check

Since our study’s clinical checklist was a physical examination form being fulfilled by the physicians, we used a panel of experts consisting of five general practitioners as external assessors to examine the Content Validity Ratio (CVR) and Content Validity Index (CVI). Experts were asked to check the essentiality and clarity of each item. Corresponding data was used in Lawshe’s formula [22] and indicated a CVI of 1 for all items except the Claudication item (CVI=0.6). The final CVR was calculated to be 0.98 from a maximum score of 1, indicating its validity. Also, a factor analysis was performed for pain questions, using the STATA MP17 software. The factor analysis revealed that the pain-related questions (current pain, best pain, and worst pain scores) are both valid and reliable measures of a common underlying pain construct, as indicated by their high factor loadings of 0.91, 0.89, and 0.80 and low uniqueness values of 0.159, 0.204, and 0.344, respectively. They consistently measure the same aspect of pain intensity.

### Data Quality Check and Analysis

TThe clinic’s IT expert was responsible for data quality checks and ensuring data accuracy and completeness. He evaluated all the recorded data every month and implemented various strategies to maintain high data quality standards, including conducting inter-rater reliability assessments to ensure consistency across the registrant nurses. He carried out periodic audits to detect any missing information and notified the registrant nurses if any of the required fields were overlooked. Furthermore, the IT expert closely monitored data for errors and discrepancies, such as incorrect entries of pain scores at best and worst conditions. Another quality control measure was to utilize generic drug names, instead of drug brands, to avoid inconsistencies during medication data entry. Before proceeding with the use of the data, the data went through a thorough revision process and revalidation to rectify any discrepancies and ensure its accuracy and integrity. The steering committee of NPC conducted comprehensive analyses of the data every six months and screened for potential errors. This multi-layered approach to data quality checks has contributed to the maintenance of high data accuracy, completeness, and consistency in MEPAIN.

### Registry System

We registered patient data in Zigorat® software, which is an online patient management system in NPC. This software was exclusively designed by Zigorat Salamat Pardazi Co., Jahrom, Iran, and launched on the server of NPC, protected by a verified firewall. It is capable of extracting and exporting data for systematical analysis, making it ideal for research purposes.

### Study Period

To establish a structured scientific database, patient enrollment for the pain registry was initiated on July 21, 2024, utilizing a standardized PRF. This instrument was designed to ensure uniform collection of clinical and demographic data pertinent to pain-related conditions. The implementation of the PRF marked the formal commencement of data registration. For the purposes of this feasibility study, data were analyzed from patients who registered up to January 22, 2025. During this period, a total of 3,903 patients who met the study’s inclusion criteria were successfully enrolled.

### Ethics Approval and Informed Consent

This study This study was performed following the Helsinki Declaration of 1964,

and its later amendments and our protocol was approved by the ethics committee of the Research Center for Neuromodulation and Pain, Shiraz University of Medical Sciences (Ethics code: IR.SUMS.REC.1403.128).

At the beginning of the study, the registrant nurse informed the patients about the purpose of this pain registry and the research nature of this project. They were reassured that only their medical records would be used for clinical and research purposes while their personal information would remain confidential. They were also warranted that they were free to withdraw from the study at any time. After the patients' thorough comprehension of the study's purpose, their consents were obtained and they were given a phone contact in case they had any questions.

### Statistical Methods

Statistical analysis was performed in two descriptive and inferential sections on the pilot phase data.

In the descriptive section, we used mean ± standard deviation (SD) for quantitative variables and frequencies (%) for qualitative variables. For inferential analysis, we utilized the Chi-Square test, independent t-test, and ANOVA for intervariable comparisons. The Chi-Square test was employed to examine relationships between categorical variables, allowing us to identify significant associations within our data.

The independent t-test was used to compare the means of two independent groups, helping to determine if there were statistically significant differences between them. ANOVA was applied to compare means across multiple groups, providing insight into whether observed differences were statistically significant across the various categories. All data were analyzed by the SPSS® software version 21 (IBM company, Texas, USA), and a P-value of less than 0.05 was considered statistically significant.

## Results

**Figure-2:**
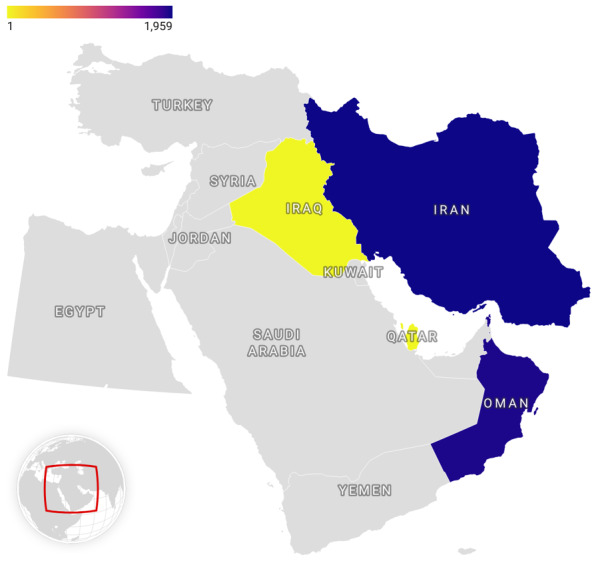
Figure 2. Map showing the geographical prevalence of chronic pain recorded during this phase of the MEPAIN initiative.

**Figure-3:**
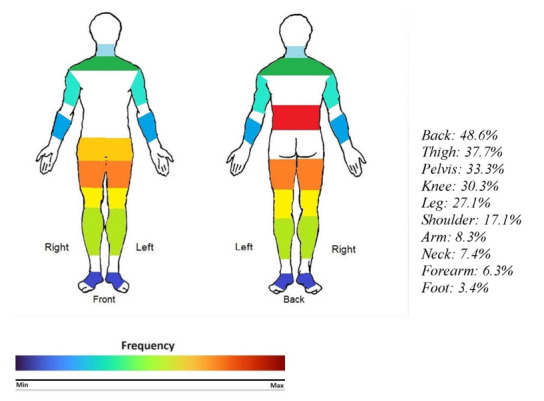
Figure 3. Top ten most frequently affected regions.

**Table:** Table3. Pain duration and intensity scores and different underlying comorbidities.

		**Pain duration** **(months)**	**P-value**	**Current pain intensity score **	**P-value**	**Pain score (best condition) **	**P-value**	**Pain score (worst condition) **	**P-value**
**Rheumatoid arthritis**	-**	36.48±0.61	0.608	5.83±0.05	0.232	4.93±0.04	0.361	8.1±0.03	0.182
	+ ^***^	37.65±4.25		5.59±0.27		4.82±0.3		7.92±0.2	
**Osteoarthritis**	**-**	36.54±0.61	0.145	5.83±0.05	0.015	4.93±0.04	0.004	8.1±0.03	0.006
	**+**	23.78±6.19		3.89±0.86		2.67±0.82		6.67±0.69	
**Hypertension**	**-**	36.69±0.72	0.318	5.83±0.05	0.337	4.94±0.05	0.304	8.11±0.03	0.338
	**+**	36.05±1.11		5.79±0.09		4.89±0.08		8.08±0.06	
**Ischemic heart disease**	**-**	36.38±0.64	0.735	5.8±0.05	0.883	4.92±0.05	0.751	8.08±0.03	0.995
	**+**	37.67±1.97		5.99±0.15		5.02±0.14		8.33±0.09	
**Diabetes mellites**	**-**	36.6±0.68	0.376	5.85±0.05	0.130	4.95±0.05	0.138	8.11±0.03	0.221
**+**	36.12±1.35		5.72±0.1		4.83±0.1		8.05±0.07	
**Hyperlipidemia**	**-**	36.41±0.67	0.630	5.82±0.05	0.591	4.94±0.05	0.216	8.08±0.03	0.888
	**+**	36.94±1.41		5.84±0.11		4.86±0.1		8.17±0.07	
**Stroke**	**-**	36.43±0.61	0.863	5.82±0.05	0.398	4.93±0.04	0.548	8.1±0.03	0.180
	**+**	42.45±6.29		5.71±0.42		4.98±0.4		7.86±0.25	
**Hypothyroidism**	**-**	36.38±0.62	0.778	5.82±0.05	0.477	4.92±0.05	0.607	8.09±0.03	0.906
	**+**	38.21±2.39		5.81±0.18		4.97±0.16		8.24±0.11	
**Hyperthyroidism**	**-**	36.57±0.61	0.152	5.82±0.05	0.408	4.92±0.04	0.761	8.1±0.03	0.887
	**+**	29.87±5.83		6.09±0.55		5.25±0.49		8.47±0.31	
**Pulmonary disease**	**-**	36.38±0.61	0.897	5.82±0.05	0.408	4.93±0.04	0.339	8.1±0.03	0.538
	**+**	41.15±3.93		5.76±0.31		4.82±0.31		8.12±0.19	
**Renal diseases**	**-**	36.65±0.62	0.144	5.82±0.05	0.494	4.93±0.04	0.431	8.09±0.03	0.880
	**+**	33.53±2.58		5.82±0.22		4.89±0.2		8.26±0.14	
Cancer	-	36.48±0.61	0.709	5.83±0.05	0.0005	4.94±0.04	0.020	8.11±0.03	0.004
	**+**	40.33±8.25		4.2±0.54		3.97±0.58		7.27±0.46	
**No disease**	**-**	36.77±0.65	0.130	5.77±0.05	0.998	4.93±0.05	0.497	8.02±0.03	1
	**+**	34.77±1.66		6.15±0.14		4.93±0.13		8.62±0.08	

^*^ Variables presented as mean±SD with 95% CI ^**^ Without the specific disease ^***^ With the specific disease

**Table:** Table4. Pain characteristics and frequency of the patients

**Variables**		**Frequency (%)**
	*Standing*	660 (16.9%)
	*Walking*	750 (19.2%)
**Aggravating factors**	*Siting*	264 (6.8%)
	*Getting up*	83 (2.1%)
	*Laying down*	42 (1.1%)
	*Standing*	5 (<0.1%)
	*Walking*	5 (0.1%)
**Alleviating factors**	*Siting*	22 (0.6%)
	*Getting up*	3 (0.1%)
	*Laying down*	371 (9.5%)
	*Paresthesia*	550 (14.1%)
**Associated symptoms**	*Claudication*	440 (11.2%)
	*Morning stiffness*	76 (1.9%)

**Table:** Table5. The frequency of involved regions across the demographic variables.

	**Total**		**Gender**			**Age**				BMI*		
		**Male**	**Female**	**P-value**	**Below 40**	**40 -60**	**Above 60**	**P-value**	**Normal**	**Overweight**	**Obese**	**P-value**
**Head**	76 (1.95)	24 (1.52)	52 (2.24)	0.109	21 (2.45)	26 (1.56)	29 (2.14)	0.231	25 (2.22)	32 (2.13)	11 (1.27)	0.25
**Face**	55 (1.41)	20 (1.27)	35 (1.51)	0.528	16 (1.87)	20 (1.2)	19 (1.4)	0.379	15 (1.33)	16 (1.07)	18 (2.08)	0.126
**Neck**	288 (7.38)	103 (6.51)	185 (7.97)	0.088	63 (7.36)	124 (7.43)	101 (7.46)	0.988	91 (8.08)	111 (7.4)	64 (7.4)	0.775
**Shoulder**	669 (17.1)	290 (18.3)	379 (16.3)	0.99	127 (16.5)	273 (16.8)	269 (17.8)	0.653	209 (16.6)	245 (16.3)	146 (16.9)	0.31
**Arm**	322 (8.25)	140 (8.86)	182 (7.84)	0.257	68 (7.94)	137 (8.21)	117 (8.65)	0.8	100 (8.88)	122 (8.13)	73 (8.44)	0.79
**Forearm**	245 (6.28)	112 (7.08)	133 (5.73)	0.086	49 (5.72)	99 (5.94)	97 (7.17)	0.245	66 (5.86)	101 (6.73)	53 (6.13)	0.645
**Hand**	72 (1.84)	37 (2.34)	35 (1.51)	0.058	13 (1.52)	24 (1.44)	35 (2.59)	0.042	17 (1.51)	33 (2.2)	17 (1.97)	0.441
**Finger**	9 (0.23)	6 (0.38)	3 (0.13)	0.109	1 (0.12)	3 (0.18)	5 (0.37)	0.401	3 (0.27)	1 (0.07)	4 (0.46)	0.145
**Chest**	14 (0.36)	7 (0.44)	7 (0.3)	0.469	1 (0.12)	8 (0.48)	5 (0.37)	0.365	3 (0.27)	7 (0.47)	4 (0.46)	0.686
**Abdomen**	37 (0.95)	17 (1.08)	20 (0.86)	0.498	5 (0.58)	15 (0.9)	17 (1.26)	0.266	12 (1.07)	17 (1.13)	8 (0.92)	0.893
**Back**	1896 (48.58)	789 (49.91)	1107 (47.67)	0.171	420 (49.07)	820 (49.16)	656 (48.48)	0.948	550 (48.85)	742 (49.43)	403 (46.59)	0.399
**Flank**	117 (3)	57 (3.61)	60 (2.58)	0.066	26 (3.04)	50 (3)	41 (3.03)	0.989	30 (2.66)	45 (3)	27 (3.12)	0.812
**Pelvis**	1300 (33.31)	531 (33.58)	769 (33.11)	0.761	280 (32.71)	571 (34.23)	449 (33.19)	0.874	383 (34.01)	504 (33.58)	273 (31.56)	0.477
**Thigh**	1471 (37.69)	607 (38.39)	864 (37.21)	0.454	305 (35.63)	665 (39.87)	501 (37.03)	0.168	422 (37.48)	575 (38.31)	315 (36.42)	0.656
**Knee**	1183 (30.3)	474 (29.98)	709 (30.53)	0.712	232 (27.1)	473 (27.92)	478 (35.33)	0.298	348 (30.91)	450 (29.98)	253 (29.25)	0.72
**Leg**	1059 (27.13)	429 (27.13)	630 (27.13)	0.998	193 (21.1)	451 (27.8)	415 (27.5)	0.456	293 (26.02)	409 (27.25)	241 (27.86)	0.631
**Ankle**	77 (2)	24 (1.52)	53 (2.28)	0.092	12 (1.4)	27 (1.59)	38 (2.81)	0.149	24 (2.13)	29 (1.93)	15 (1.73)	0.816
**Foot**	132 (3.38)	58 (3.67)	74 (3.19)	0.414	35 (4.09)	48 (2.88)	49 (3.62)	0.212	43 (3.82)	49 (3.26)	29 (3.35)	0.728
**Toe**	63 (1.61)	24 (1.52)	39 (1.68)	0.694	12 (1.4)	30 (1.8)	21 (1.55)	0.764	19 (1.69)	23 (1.53)	19 (2.2)	0.486

* Includes missing data. ** Chi-Square test *** Variable frequencies are presented as numbers (%).

**Table:** Table6. The frequency of different interventions among gender, age, and BMI variables.

**Recommendations**	**Total**		**Gender**			**Age**				**BMI**		
		**Male**	**Female**	**P-value**	**Below 40**	**40 -60**	**Above 60**	**P-value**	**Normal**	**Overweight**	**Obese**	**P-value**
**Transforaminal epidural Steroid injection (TFESI)**	1359 (34.8)	552 (34.91)	807 (34.75)	0.907	316 (36.96)	581 (34.3)	462 (34.15)	0.333	386 (34.28)	536 (35.71)	302 (34.91)	0.746
**Caudal epidural steroid injections**	1336 (34.2)	531 (33.59)	805 (34.67)	0.493	303 (35.44)	582 (34.3)	451 (33.33)	0.592	377 (33.48)	523 (34.84)	302 (34.91)	0.722
**Radiofrequency (RF) ablation**	1068 (27.4)	441 (27.89)	627 (27)	0.532	257 (30.06)	442 (26.09)	369 (27.27)	0.105	303 (26.91)	410 (27.32)	241 (27.86)	0.894
**Local Ozon injection**	926 (23.7)	371 (23.47)	555 (23.9)	0.762	217 (25.38)	411 (24.26)	298 (22.03)	0.155	260 (23.09)	354 (23.58)	217 (25.09)	0.565
**Local Hyaluronic acid injection**	777 (19.9)	304 (19.23)	473 (20.37)	0.386	176 (20.58)	352 (20.78)	249 (18.4)	0.226	215 (19.09)	299 (19.92)	178 (20.58)	0.706
**Oral medications**	653 (28.2)	386 (24.41)	599 (25.8)	0.335	196 (22.92)	454 (26.8)	335 (24.76)	0.092	298 (26.47)	377 (25.12)	208 (24.05)	0.459
**Local Steroid injection**	461 (11.8)	188 (11.89)	273 (11.76)	0.893	102 (11.93)	192 (11.33)	167 (12.34)	0.688	138 (12.26)	178 (11.86)	95 (10.98)	0.676
**Epidural steroid injection**	390 (10)	150 (9.49)	240 (10.34)	0.389	94 (10.99)	157 (9.27)	139 (10.27)	0.357	113 (10.04)	151 (10.06)	84 (9.71)	0.959
**Physiotherapy**	181 (4.6)	61 (3.86)	120 (5.17)	0.057	39 (4.56)	83 (4.9)	59 (4.36)	0.775	48 (4.26)	58 (3.86)	50 (5.78)	0.087
**Surgery**	181 (4.6)	72 (4.55)	109 (4.69)	0.841	34 (3.98)	82 (4.84)	65 (4.8)	0.581	45 (4)	77 (5.13)	42 (4.86)	0.384
**Platelet-rich plasma (PRP) injection**	96 (2.5)	43 (2.72)	53 (2.28)	0.385	15 (1.75)	36 (2.13)	45 (3.33)	0.033	30 (2.66)	34 (2.27)	17 (1.97)	0.58
**Lifestyle modification**	71 (1.8)	23 (1.45)	48 (2.07)	0.161	8 (0.94)	32 (1.89)	31 (2.29)	0.065	21 (1.87)	24 (1.6)	19 (2.2)	0.577
**Greater occipital nerve block**	55 (1.4)	23 (1.45)	32 (1.38)	0.84	11 (1.29)	24 (1.42)	20 (1.48)	0.933	17 (1.51)	20 (1.33)	11 (1.27)	0.887
**Percutaneous laser disc decompression (PLDD)**	23 (0.6)	6 (0.38)	17 (0.73)	0.158	8 (0.94)	6 (0.35)	9 (0.67)	0.175	10 (0.89)	7 (0.47)	4 (0.46)	0.319
**Ozone autohemotherapy**	8 (0.2)	2 (0.13)	6 (0.26)	0.372	0 (0)	5 (0.3)	3 (0.22)	0.294	2 (0.18)	2 (0.13)	2 (0.23)	0.856
**Interlaminar epidural steroid injections**	4 (0.1)	2 (0.13)	2 (0.09)	0.099	0 (0)	3 (0.18)	1 (0.07)	0.386	2 (0.18)	2 (0.13)	0 (0)	0.489
**Brace**	3 (0.1)	1 (0.06)	2 (0.09)	0.801	1 (0.12)	1 (0.06)	1 (0.07)	0.882	0 (0)	2 (0.13)	1 (0.12)	0.485
**Intradiscal ozone injection**	1 (0.02)	1 (0.06)	0 (0)	0.225	1 (0.12)	0 (0)	0 (0)	0.168	1 (0.09)	0 (0)	0 (0)	0.35

^*^ Independent t-test or ANOVA with Tukey post hoc analysis.

A Total of 3903 eligible medical records were registered in MEPAIN (59.5% female and 40.5% male). The mean age was 53.54 ± 14.78 years with the majority (43.4%) of the patients being middle-aged adults (40 to 60 years). Most of the participants (60.7%) were obese or overweight while only 28.8% had normal body mass index (BMI). About 10% refused to report their height and weight. The mean BMI was 27.4± 4.52 kg/m2.

Approximately half of the MEPAIN registry participants were Iranian (50.2%), while 49.45% were from Oman. A smaller proportion of patients originated from Iraq, Bahrain, and Qatar. The subjects’ pre-existing medical conditions are listed in table-1.

Mean pain duration, intensity scores, and the frequencies of different pain diagnoses are represented and compared among demographic variable groups in Table-2. Bold variables indicate a significant difference.

As shown in the above table the most common pain diagnoses were lumbar radiculopathy,‎ osteoarthritis, and discogenic pain. There were 547 (14.01%) patients whose chronic pain was idiopathic. The most common diagnosis was lumbar radiculopathy among all gender, age, and nationality groups.

The intervariable comparisons revealed that the prevalence of osteoarthritis and carpal tunnel syndrome were significantly higher in females (P<0.01 for both) while there were no significant differences in pain duration and intensity indices between males and females (P>0.05).

Significant age-related differences were also observed in the prevalence of pain diagnoses. All conditions were significantly more prevalent in older age groups except lumbar radiculopathy and discogenic pain.

Figure-2 illustrate Regional distribution of chronic pain derived from the current phase of the MEPAIN project. Regarding nationality, Iranians had higher pain intensity scores at worst conditions compared with Omanis, but the prevalence of discogenic pain, spinal stenosis, carpal tunnel syndrome, and failed back surgery syndrome were significantly higher among the Omani patients.

Table-3 demonstrates pain duration and intensity scores with different underlying comorbidities.

Patients with osteoarthritis and cancer had significantly lower pain intensity scores compared with patients without these conditions. Other pain features are depicted in Table-4.

As shown above, the most common aggravating factor was walking (19.2%) and the most common alleviating factor was lying down (9.5%). However, no aggravating or alleviating factor was reported in 2104 and 3831 participants, respectively. Among the questioned accompanying symptoms, paresthesia was the most common accompanying symptom (14.1%). None of the patients reported symptoms such as weight loss, decreased appetite, or night sweating. Regarding the number of involved areas, in 1257 (32.6%) patients only one area was involved while in 2127 (54.5%) patients two to four areas, and in 302 (7.7%) patients more than four areas were involved. The rest of the patients didn’t specify the number of their involved areas. Table-5 demonstrates different locations of chronic pain in patients.

As shown in Table-5, back (48.6%), Thigh (37.7%), and pelvis (33.3%) were the most common regions involved, respectively. Back was the most commonly involved region across all gender, age, and BMI groups. The pain drawing scale shown in figure-3 highlights the top ten most frequently affected regions.

The ten most commonly involved regions didn’t show a significant difference among different age, gender, and BMI groups; however, hand pain was significantly more prevalent in patients above 60 years old (p-value: 0.042).

On physical examination, tenderness of facet joints, sacroiliac joints, and spinous processes were observed in 3.3%, 3.2%, and 0.3% of patients, respectively. Considering the range of motion, painful flexion was the most common abnormal finding (2.3%), followed by painful extension (1.5%), flexion restriction (0.5%), and extension restriction (0.3%). Moreover, 3.3% and 2.3% of patients displayed abnormal heel and toe walking tests, respectively.

Table-6 illustrates the interventions that were done for the patients in NPC, the most common of which was transforaminal epidural steroid injection (TFESI). It was also the most commonly utilized intervention among all gender, age, nationality, and BMI groups. Out of all the patients, most underwent at least one procedure while 653 (28.2%) were only prescribed oral medications and 71 (1.8%) were advised to make lifestyle changes.

Chronic pain is highly prevalent and causes significant distress and impaired function. [13] To address this widespread issue, pain registries play a crucial role in improving the understanding, management, and treatment of this condition. By systematically collecting and analyzing data from diverse patient populations, these registries enable researchers and healthcare providers to identify patterns and trends in pain experiences and treatment outcomes. For example, the insights gained from pain registries can be used to provide evidence-based clinical guidelines, enhance personalized treatment plans, and facilitate the development of new therapeutic interventions. Moreover, they provide a valuable resource for tracking the long-term efficacy and safety of pain treatments, helping to identify best practices and areas needing improvement. Ultimately, the impact of pain registries extends beyond individual patient care, influencing public health policies and advancing the field of pain research.

Globally, various pain registries have been developed to address chronic pain. Examples include the low back pain registry of PRECISION (established in 2016), which is a biopsychosocial repository of data on patients with lower back pain [22]; the Oslo Pain Registry (OPR) in Norway [23]; the Greek Neuropathic Pain Registry (Gr.NP.R.) [24]; the Swedish Quality Registry for Pain Rehabilitation (SQRP) [25]; the Quebec Pain Registry (QPR) in Canada [26]; the Danish clinical registry of chronic back pain (SpineData) [27]; and the German Pain Practice Registry [28]. These registries serve as critical tools for understanding and managing chronic pain across different populations and regions.

The utility of pain registries is further demonstrated by their application in clinical research. Several studies have utilized data from these registries to extract detailed information on chronic pain characteristics and design various clinical trials. For instance, a very recent study used data from the QPR to compare chronic pain treatment between patients in remote and non-remote areas of Quebec [29]. Similarly, in 2023, Halvorsen et al. designed a clinical trial using patient data from the OPR to assess the feasibility of titrating or tapering opioid medications through a nurse-led telephone follow-up for chronic pain patients [30]. Another example is a randomized trial in 2020 that utilized the Danish SpineData to evaluate the efficacy of spinal manipulation at segments of stiffness or pain sensitivity in improving pain intensity in patients with chronic low back pain [31]. These studies highlight the pivotal role of pain registries in advancing evidence-based pain management.

To the best of our knowledge, MEPAIN is the first chronic pain registry in the Middle East, launched for patients referred from different countries in the region to a center in Iran. Unlike most pain registries, which are limited to a single center or nation, MEPAIN is multinational despite being a single-center data repository. This unique feature is due to medical tourism in the Middle East, with the Nab pain clinic in Shiraz, Iran, serving as a destination for patients across the region. This multinational aspect provides a valuable opportunity to obtain generalized and practical information on chronic pain in a diverse population.

Among the pain registries highlighted, QPR, OPR, and SQRP were dedicated to chronic pain patients. Interestingly, lumbar radiculopathy was the most common pain diagnosis among MEPAIN and QPR patients, while OPR and SQRP did not delve into the causes of chronic pain. Notably, all these registries utilized a 10-score scale for pain intensity measurement. The average pain intensities were 5, 6.71, 6.02, and 6.8 in MEPAIN, QPR, OPR, and SQRP patients, respectively. A more detailed comparison is demonstrated in Table-7.

The findings from our study have significant implications for understanding the demographic and clinical patterns of chronic pain conditions. For example, the predominance of lumbar radiculopathy across all groups suggests a widespread need for targeted interventions for this condition. Preventive measures such as maintaining proper posture, engaging in regular physical exercise, and ergonomic modifications in the workplace can help reduce its incidence. Additionally, the higher prevalence of osteoarthritis and carpal tunnel syndrome in females highlights the importance of gender-specific approaches in pain management and treatment. Preventive strategies for these conditions may include weight management, hand and wrist exercises, and early intervention with ergonomic tools. Furthermore, the fact that most conditions were more common in older age groups underscores the impact of aging on pain development and the necessity for age-appropriate pain management strategies. For older adults, maintaining an active lifestyle, engaging in strength and flexibility exercises, and regular medical check-ups can serve as preventive measures. Moreover, the frequent involvement of the back region in chronic pain patients indicates a critical area for therapeutic focus. Preventive measures such as core strengthening exercises, proper lifting techniques, and avoiding prolonged sitting can help mitigate back pain. Finally, the significant prevalence of hand pain in patients over 60 points to the need for specialized care for older adults suffering from this symptom. Hand exercises, ergonomic adjustments, and early medical intervention can prevent the worsening of hand pain in this population. Overall, these findings emphasize the necessity for personalized and demographic-specific approaches in the management, treatment, and prevention of chronic pain.

## Discussion

**Table:** Table7. Comparison of the pain registries.

**Name of Registry**	**Location**	**Methods**	**Sample Size**	**Data Collection Period **	**Challenges Noted**	**Registry Management**
**Oslo University Hospital Pain Registry (OPR) [23] **	Oslo, Norway	electronic data entry before consultations	1,712	2015 - 2017	Only local data, varying clinician data entry	Research, Oslo University Hospital
**Quebec Pain Registry (QPR) [26] **	Quebec, Canada	Web-based registry, nurse-administered questionnaires	9,363	2008 - 2014	Ensuring data completeness, varying patient participation	Quebec Pain Research Network
**SpineData [27] **	Southern Denmark	Internet-based system, patient and clinician electronic questionnaires, linked with national registries	35,466	January 1, 2011 - July 17, 2014	Variability in clinician adherence to standardized methods, missing electronic data from paper forms	Medical Department of the Spine Centre of Southern Denmark, Hospital Lillebaelt
**Greek Neuropathic Pain Registry (Gr.NP.R.) [24] **	Greece	Multicenter registry, patient data on demographics, medical history, pain type, and treatments	5980	2016 - 2020	Coordination across multiple centers, ensuring adherence to national guidelines	Hellenic Society of Pain Management and Palliative Care
**Nab Pain Registry (NPR)**	Persian Gulf countries	Electronic data collection, demographic and clinical data, follow-up reports	3,903	March 21, 2022 - September 22, 2022	Data accuracy and completeness, inter-rater reliability among registrant nurses	Research Center for Neuromodulation and Pain, Nab Pain Clinic, Shiraz University of Medical Sciences

Chronic pain can arise from various causes; however, in some cases, no pathologic basis can be found, a condition referred to as chronic idiopathic pain syndrome. In these cases, no organic explanation can be identified despite a thorough medical assessment. These patients often see multiple doctors and overutilize healthcare facilities, imposing a significant burden on the healthcare system. While many studies have demonstrated the association between this type of chronic pain and psychological conditions such as depression and anxiety, the causal relationship has not yet been proven [32]. The development of a pain registry in the region allows us to better identify these individuals and investigate the efficacy of different treatment modalities in this population. This, in turn, enables us to address their needs more efficiently and reduce the burden on healthcare systems.

In this pain registry, we paid careful attention and documented the findings of physical examination as the possible underlying etiology of some chronic pain disorders can often be identified by the hints left in physical examination [33]. This emphasis on physical examination is crucial because the presence of multiple tender points, for instance, favors fibromyalgia [34], while spinal tenderness or limited range of motion yields additional clues about the type of lower back pain [35][36][37]. Moreover, findings of some clinical tests also carry a prognostic value. Although the evidence remains controversial, some studies have shown an association between these findings and various outcome measures. For example, Flynn et al. demonstrated that limited hip internal rotation implicates a worse outcome [38]. Similarly, multiple studies revealed that abnormal neurological signs indicate poorer outcomes in pain, disability, return to work, and global improvement [39][40][41]. These findings are not only diagnostic but also therapeutic, as they assist clinicians in choosing a management strategy and individualizing treatment plans [42]. Therefore, developing a systematic pain registry will aid physicians in using this information to predict treatment outcomes and, consequently, choose more appropriate treatment options based on prognosis.

While physical examination is invaluable, it is not definitive on its own. Physical examination alone cannot conclusively diagnose chronic pain conditions, but it provides valuable information and helps guide the choice of imaging tests to identify possible causes of pain. As such, physical examination findings should be interpreted in conjunction with other clinical data [33][43]. This integrated approach ensures a more accurate diagnosis and effective treatment plan.

Complementing physical examination, diagnostic imaging (such as X-rays, MRI, or CT scans) plays a critical role in chronic pain assessment [44]. These imaging modalities help visualize anatomical structures, identify structural abnormalities, and rule out specific pathologies. For example, imaging can reveal herniated discs, degenerative changes, or inflammatory processes [45]. However, it’s essential to recognize that imaging findings do not always correlate directly with the presence or severity of pain. Some individuals may have abnormal imaging results without experiencing significant pain, while others may have pain despite normal imaging findings [43]. Therefore, the optimal approach involves integrating both physical examination findings and diagnostic imaging results [46]. Clinicians must consider the patient’s history, symptoms, and physical examination findings alongside imaging data. This combined assessment allows for a more comprehensive understanding of the underlying pain mechanism, and treatment decisions should be based on the overall clinical picture rather than relying solely on imaging results [44].

In summary, while physical examination and diagnostic imaging provide valuable insights, a holistic approach that considers both aspects is crucial for accurate diagnosis and effective management of chronic pain disorders [33]. This is where MEPAIN plays a pivotal role, as it allows systematic registration of data obtained from the patient’s history, physical examination, and imaging workups. By consolidating this information, MEPAIN enhances the ability to tailor treatment plans to individual patient needs.

In the existing literature and relevant guidelines, various treatment options have been recommended for chronic pain management, including lifestyle modification, physical therapy and exercise, pharmacological interventions, corticosteroid and PRP injections, Ozone Therapy, etc. [14][47][48] These interventions have demonstrated variable efficacies in decreasing pain, depending on the underlying condition and patient factors [49][50]. In line with this, we have attempted to offer scientifically approved treatment options to this population. Given the multifactorial nature of chronic pain and diverse underlying pathologies, multiple treatment modalities are often required to provide significant relief [51]. Ultimately, the decision must be made considering patient preferences, values, and underlying conditions to create a personalized treatment plan [50].

MEPAIN has the potential to provide a context that helps us determine and recommend the most appropriate treatment modalities to the patients.

Looking ahead, the future is optimistic, especially given the ongoing measures that aim to improve the efficiency of MEPAIN as a research resource. A principal strategy is to expand the scope of outcome measures beyond the pain index, to include disability and quality of life indices as well. Additionally, a systematic approach is planned to be introduced by conducting psychological evaluations of patients, which is crucial in understanding pain development and prognosis, especially in idiopathic chronic pain syndrome. This holistic perspective aims to explore patients' unmet needs, bringing not only new knowledge but also the realization of proper and tailored care delivery.

Another critical dimension of our future course is to enhance the capacity of data collection and grow alliances with other sources, such as electronic health records, imaging, and laboratory reports.

Such an empowered method of action is intended to facilitate patient evaluation processes and avoid excessive workups. Furthermore, in the future, we will record adverse events following procedures, such as bloody puncture during epidural access and post-procedural hematomas, which are typically reported during follow-ups.

By expanding this pain registry, we can achieve an enormous data repository that will provide the foundation for further evidence-based research. Using this information, we can develop guidelines for chronic pain management tailored to our local setting, enabling a patient-centered approach for dealing with chronic pain syndromes. Our future studies will aim to explore the long-term outcomes of chronic pain interventions and compare our results with other pain registries. By doing so, our registry will contribute to the global research on pain control.

### Limitations

The MEPAIN, like other similar registries, faces certain limitations regarding the data collection methodology. Although our goal is to include patients from across the Middle East, the pilot phase primarily consists of Iranian and Omani participants.

We are currently working to expand the registry by incorporating data from additional clinics in the region.

One significant limitation stem from the inherently subjective nature of pain and the outcomes following treatment interventions, as these rely heavily on patient-reported information. Patient responses can be influenced by various interpersonal and intrapersonal factors, which can affect the accuracy of the data. This becomes particularly critical when considering the possibility of missing data during follow-ups, as patients may overestimate positive responses to treatment. [22]

One additional constraint is that our recorded data only includes the physical examinations and diagnostic workups conducted by our specialists at our center. However, it is important to note that many patients, referred to our clinic, have previously been evaluated by other physicians and undergone diagnostic workups, but we did not have access to those medical records. Consequently, the inclusion of diverse diagnostic workups in our data would not achieve the necessary level of saturation for statistical evaluation.

Another pitfall of this study and any other pain registry is the questionable reliability of the data from the patients with cognitive impairment, including patients with Alzheimer's disease or individuals with intellectual disability. They are unable to comprehend the concept of the questions and communicate thoroughly about their pain experience. Thus, they can't provide reliable information. Addressing the challenge of pain assessment in this population requires a tailored approach and implementation of several strategies and tools. Healthcare providers can observe behavioral cues, such as facial expressions, body language, vocalizations, and changes in activity level, to infer pain levels. Caregivers or family members who are familiar with the patient's behaviors and communication patterns can also provide useful information about the patient's pain experience. Overall, individualized approaches are necessary to meet each patient’s unique needs.

## Conclusion

MEPAIN was developed to systematically collect data from patients referred to NPC across the Middle East, with the primary aim of documenting pain characteristics and treatment interventions. This registry serves both clinical and research purposes by providing reliable data to support evidence-based treatment decisions and personalized pain management.Moreover, the MEPAIN is open to integrating data from other clinics, provided the data is collected by the registry's standard protocols. With its robust framework, MEPAIN has the potential to become the official chronic pain data repository for the region, contributing to valuable, evidence-based insights into chronic pain statistics and management strategies. In this study, we introduced the MEPAIN and analyzed significant demographic data from the pilot phase, which will inform a more precise, patient-centered approach in our local healthcare settings moving forward.

## Conflicts of Interest

A. Karimi, serving as the editor of GMJ, did not participate in the review process for this manuscript. An independent editor oversaw all editorial decisions to ensure an unbiased evaluation. The other authors declare that they have no conflicts of interest related to this work.
